# Out of hospital cardiac arrest in Western Sydney—an analysis of outcomes and estimation of future eCPR eligibility

**DOI:** 10.1186/s12873-022-00587-8

**Published:** 2022-02-28

**Authors:** Pramod Chandru, Tatum Priyambada Mitra, Nitesh Dutt Dhanekula, Mark Dennis, Adam Eslick, Natalie Kruit, Andrew Coggins

**Affiliations:** 1 Emergency Department, Westmead Hospital, Sydney, NSW 2145 Australia; 2 Westmead Clinical School, The University of Sydney, Sydney, Australia; 3 Department of Cardiology, Royal Prince Alfred Hospital, Sydney, Australia; 4 Department of Anaesthetics, Westmead Hospital, Sydney, Australia; 5 Simulated Learning Environment for Clinical Training (SilECT), Sydney, Australia

**Keywords:** Out of Hospital Cardiac Arrest, eCPR, ECMO, Utstein, Service Planning

## Abstract

**Background:**

Refractory out of hospital cardiac arrest (OHCA) is associated with extremely poor outcomes. However, in selected patients extracorporeal cardiopulmonary resuscitation (eCPR) may be an effective rescue therapy, allowing time treat reversible causes. The primary goal was to estimate the potential future caseload of eCPR at historically 'low-volume' extracorporeal membrane oxygenation (ECMO) centres.

**Methods:**

A 3-year observational study of OHCA presenting to the Emergency Department (ED of an urban referral centre without historical protocolised use of eCPR. Demographics and standard Utstein outcomes are reported. Further, an a priori analysis of each case for potential eCPR eligibility was conducted. A current eCPR selection criteria (from the 2-CHEER study) was used to determine eligibly.

**Results:**

In the study window 248 eligible cardiac arrest cases were included in the OHCA registry. 30-day survival was 23.4% (*n* = 58). The mean age of survivors was 55.4 years. 17 (6.8%) cases were deemed true refractory arrests and fulfilled the 2-CHEER eligibility criteria. The majority of these cases presented within “office hours” and no case obtained a return of spontaneous circulation standard advanced life support.

**Conclusions:**

In this contemporary OHCA registry a significant number of refractory cases were deemed potential eCPR candidates reflecting a need for future interdisciplinary work to support delivery of this therapy.

## Background

Survival following out of hospital cardiac arrest (OHCA) has been historically poor, with limited improvements in outcomes over time reported in many settings [1, 2]. A limited number of jurisdictions, such as King County in Washington State, have reported step-wise improvements in OHCA outcomes as the result of coordinated interagency and public health initiatives [3, 4]. Rates of achieving return of spontaneous circulation (ROSC) following advanced life support (ALS) in a prehospital setting vary depending on local geography, public policy, bystander actions and logistical factors and access to healthcare resources [5–7]. In spite of high quality cardiopulmonary resuscitation (CPR) and best practice ALS, a proportion of OHCA cases are transported to hospital without ROSC and are termed ‘*refractory*’. In these refractory cases, extracorporeal membrane oxygenation (ECMO), or “eCPR’’ may be a management option as a bridge to definitive therapy [8, 9].

In carefully selected OHCA populations observed improvements in patient outcomes when compared to standard ALS protocols have been dramatic with multiple trials showing eCPR for OHCA and refractory ventricular fibrillation (VF) significantly improved survival to hospital discharge [10–13].

While mechanical CPR (m-CPR) does not show stand-alone survival benefit in randomised trials [14], it is most likely non-inferior to manual chest compressions and allows for effective on-going resuscitation during transport. Use of M-CPR, therefore may help facilitate earlier cannulation of potentially viable eCPR patients on arrival to hospital [6, 15, 16]. Implementing these systematic changes requires coordination from all disciplines of critical care and a larger whole of hospital approach to ensure appropriate clinician support and resource allocation. Furthermore, coordination with prehospital services is key in ensuring appropriate patient selection and notification for all potential ECMO cases.

In order to further the knowledge and understanding of the application of advanced OHCA treatment strategies in an Australasian setting, we set out to prospectively assess the baseline and future management of a contemporary OHCA cohort in a local health district (LHD) with a historically limited use of ECMO for in-patients and no history of use of eCPR in the ED [1]. Using this prospective OHCA database we applied an evidence based selection criteria to determine caseloads that might be experienced by future eCPR teams.

## Methods

### Study setting and eligibility criteria

This project was conducted at Western Sydney Local Health District (WSLHD) in Sydney, Australia. The tertiary centre in this network, Westmead Hospital, is an urban, university-affiliated hospital with a pre-pandemic ED census of 79,000 annual presentations. Protocols were approved by the WSLHD Human Research Ethics Committee (*HREC Code 5529*). The prospective observational database used for this study included consecutive ED OHCA presentations from 2016–2019. Utstein reporting methods were used for the data collection [17]. The data points were collected by a single investigator (PC) with assistance from trained data managers. In WSLHD Westmead Hospital in the major tertiary referral hospital within the district and services a population of 2,000,000 people however not all cardiac arrests in the local health district are referred to Westmead, leading to limitations around generalisability.

**Fig. 1:**
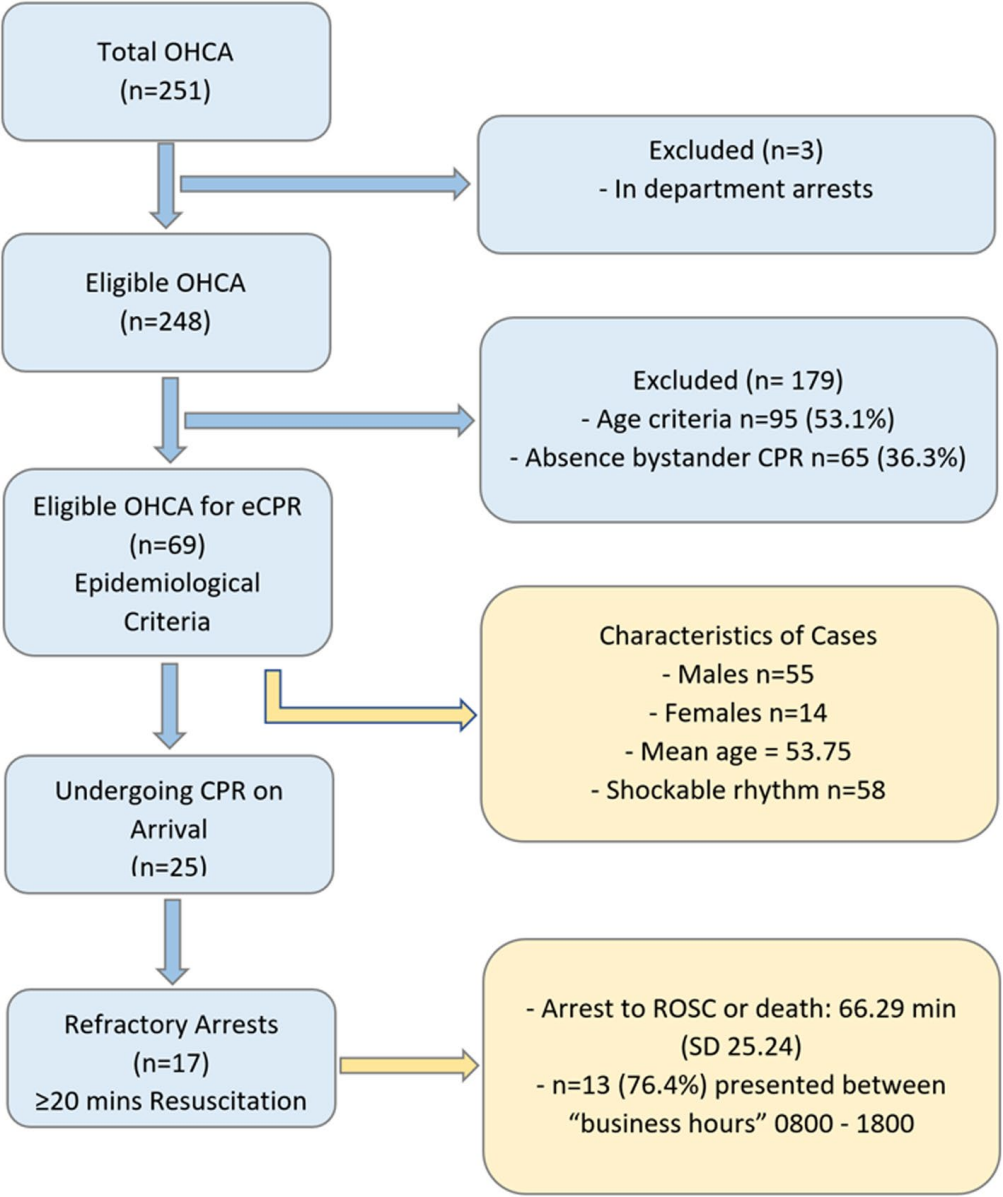
Out of Hospital Cardiac Arrest (OHCA) STARD Diagram

The a priori inclusion criteria were: (1) OHCA age ≥ 16 years; (2) eMR notes accessible; (3) *OHCA or cardiac arrest in ED within 1-h of arriva*l. Exclusion criteria were *(1) In-patient cardiac arrests (defined as cardiac arrest* > *1-h following presentation to ED); (2) Paediatric cases age* < *16 years.* These criteria were applied to ED Electronic Medical Record (eMR) presentation lists (generated on a weekly basis over a 3-year period by an ED data manager). Eligible OHCA were manually evaluated using paper forms with additional data points obtained from eMR and state ambulance records. eCPR eligibility criteria (Table 1) used by the 2-CHEER study were used to retrospectively match the likely use of eCPR in the cohort of OHCA patients (Fig. 1) [1, 14].

**Table 1:** Published 2-CHEER Study eCPR Eligibility Criteria

Inclusion criteria	Exclusion criteria
• Patients with OHCA refractory to CCPR were eligible for ECPR if they were aged 12–70 years, *AND*	Patients were excluded if there was active bleeding, if it was known that the patient did not want to receive invasive resuscitation, or if the patient had a pre-existing comorbidity and/or functional limitation such that it would prevent a future return to independent life
1. The cardiac arrest was likely to be of primary cardiac or respiratory cause (including myocardial depression secondary to hypothermia or drug effects)	
2. The cardiac arrest was witnessed AND chest compressions commenced within 10 min	
3. Initial cardiac rhythm of ventricular fibrillation or ventricular tachycardia	
4. Immediate availability of a mechanical cardiopulmonary resuscitation (CPR) device with paramedic staff; *AND*	
1. The cardiac arrest duration (collapse to arrival time at the ED less than 60 min	

Source [14]

### Analysis Plan

The primary outcome measure (Fig. 1) was a manual point estimate of OHCA cases matching eCPR eligibility criteria (Table 1). For this outcome, cases were matched retrospectively by two reviewers using an a priori plan. Discrepancies in group allocation between reviewers were resolved by consensus after further review. We defined a refractory arrest as a patient receiving CPR on arrival to the ED with an arrest to ROSC time or an arrest to death time of ≥ 20 min [18]. Secondary outcomes included demographic characteristics based on modified Utstein methods, 30-day all-cause mortality, assessment of factors affecting the timing of ROSC [17]. Hospital intervention including cardiac catheterisation and use of M-CPR were also recorded prospectively. Means, standard deviations (SD), medians and interquartile ranges (IQR) are used to report descriptive data as appropriate for parametric and non-parametric distributions respectively and Chi-squared tests were used for significance tests of reported comparative outcome data.

Over the course of the study data from NSW Ambulance registry demonstrates that 27% of resuscitation attempts initiated by pre hospital services survived to the emergency department and this therefore represents an element of selection bias through the use of termination of resuscitation (TOR) protocols in the field. This however reflects a population of patients that would not be eligible for eCPR interventions as per the 2CHEER criteria adopted in the district. Current termination of resuscitation guidelines suggest that if ROSC is not achieved in 20 min and the patient is not in a shockable rhythm a decision may be made to cease resuscitation efforts. In 2019, NSW Ambulance registry data demonstrated that 50% of patients had resuscitation efforts ceased on scene with very few patients (2%) surviving ED admission if they were transported with CPR en route. Again this reflects a selection bias that does need to be taken into consideration when interpreting the data regarding eligibility of all cardiac arrests presenting to Westmead Emergency, however for those patients persistently in a non shockable rhythm eCPR is unlikely to be an offered treatment under current guidelines.

## Results

**Fig. 2:**
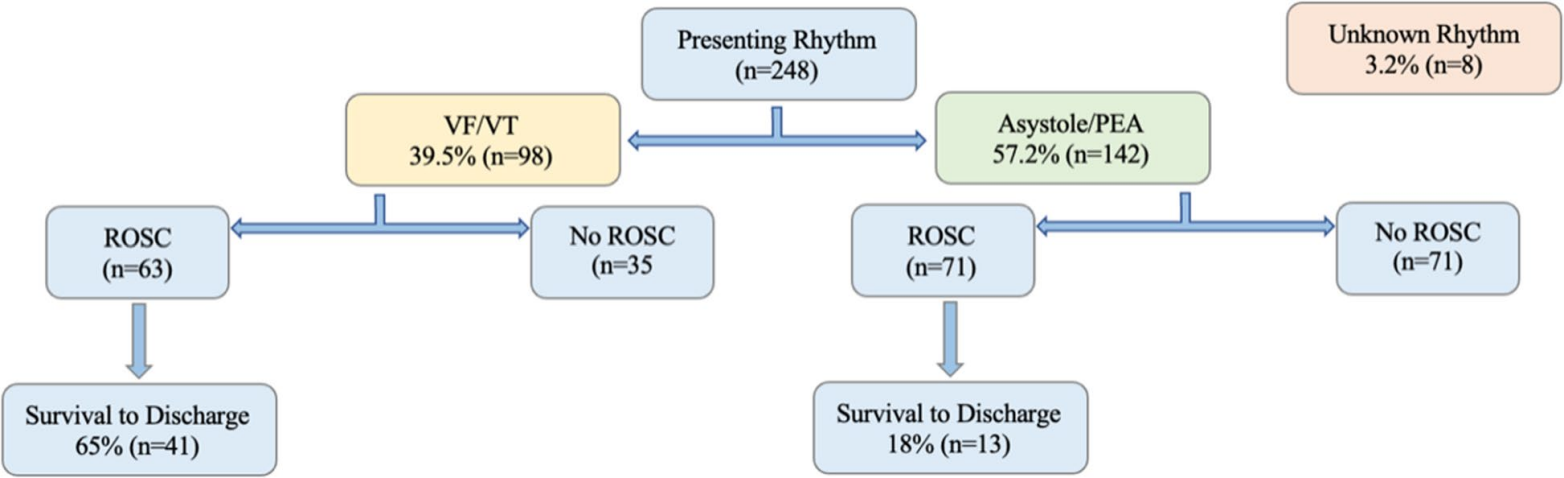
Survival to Discharge by Presenting Rhythm and eCPR Inclusion Criteria

Within the study window there were 193,750 ED presentations and 251 OHCA cases identified. In emergency department patient arrests (*n*  =  3) were excluded from the analysis (Fig. 1). Overall reported survival (30-days) was 23.4% (*n*  =  58). The mean age of survivors was 54.5 years (IQR 21.25). Bystander CPR was reported in 175 cases (Table 2). Arrests were witnessed in 196 OHCA (79.0%) cases and bystander CPR was presented in 89% of cases. Both witnessed and the presence of bystander CPR was associated with a significant increase in likelihood of survival bystander, CPR (26.3% vs 14.3%, *p  *=  0.043) and In comparing witnessed and unwitnessed arrests there was a significant survival likelihood in witnessed arrests (10.0% v 27.0%; *p*  <  0.001). Ventricular fibrillation (VF) and ventricular tachycardia (VT) were reported in 98 cases (38.3%) (Fig. 2). Asystole and pulseless electrical activity (PEA) accounted for 142 cases (57.2%) and the remaining 8 cases being of undocumented rhythm (Table 3).

**Table 2:**
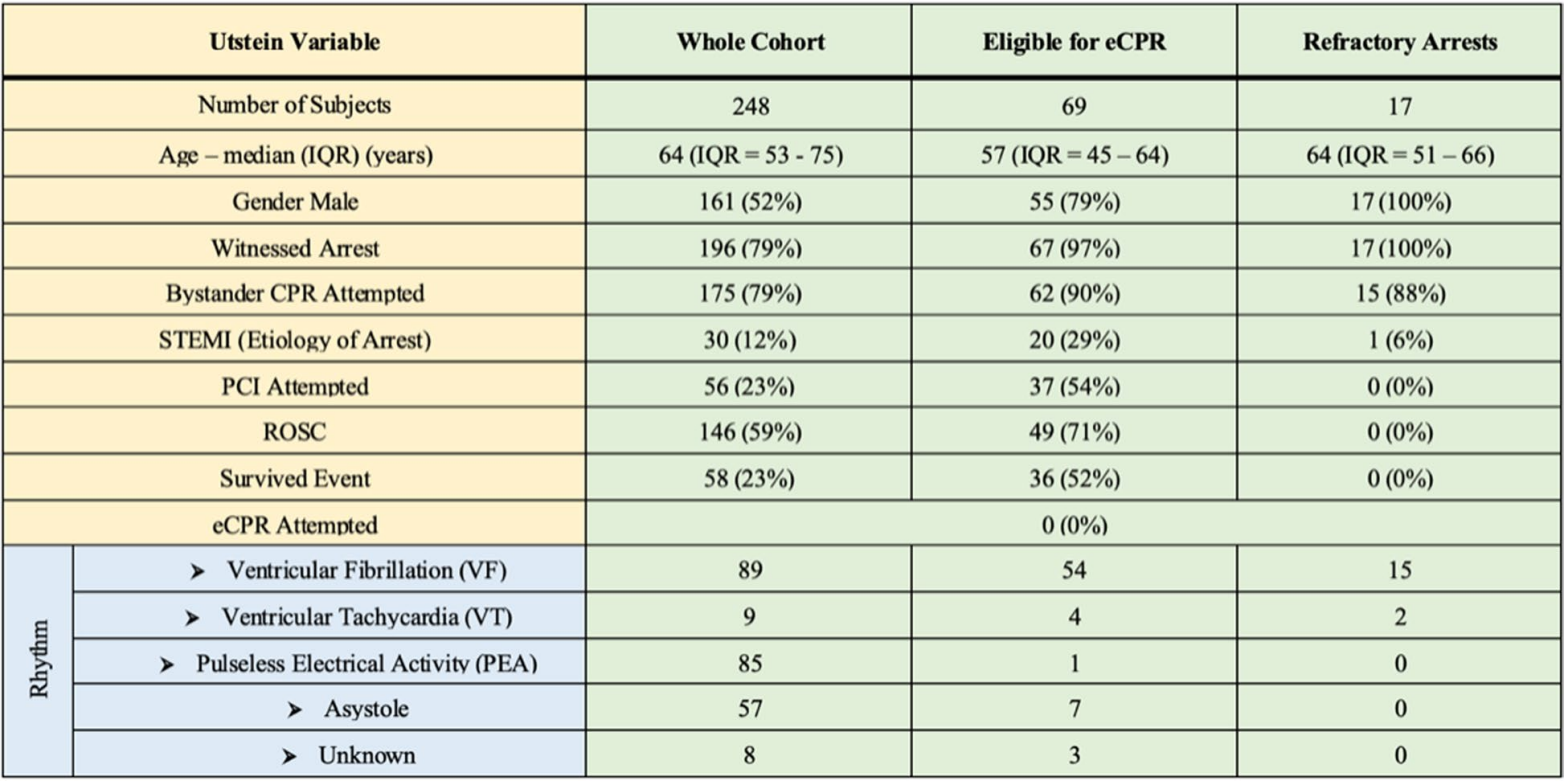
Characteristics of Out of Hospital Cardiac Arrest Patients

**Table 3:** Survival to Discharge by Presenting Rhythm and eCPR Inclusion Criteria

Primary Rhythm	Total Number	ROSC (n/%)	Survival (n/%)
Ventricular Fibrillation (VF)	89	57 (64%)	38 (43%)
Ventricular Tachycardia (VT)	9	6 (66%)	3 (33%)
Pulseless Electrical Activity (PEA)	85	47 (55.3%)	8 (9.4%)
Asystole	57	24 (42.1%)	5 (8.7%)

Witnessed OHCA accounted for 196 (79.0%) cases. Of patients receiving bystander CPR, 62.3% (n = 96) had ROSC with 26.3% (*n*  =  47) surviving to hospital discharge. Of witnessed arrests who did not receive bystander CPR (*n*  =  42) the rate of ROSC was 57.1% (*n*  =  24) and survival to hospital discharge was 16.6% (*n*  =  7). In unwitnessed arrests (*n*  =  50) 20 patients experienced ROSC with 5 surviving to hospital discharge.

Of all patients included in the study fifty-five patients (22.2%) underwent cardiac catheterisation within 24-h of ED arrival. No patients were reported to have received pre-hospital M-CPR. 23 patients taken for PCI had a documented diagnosis of ST elevation myocardial infarction. Of the 55 patients taken for percutaneous coronary intervention (PCI), 43 (78.1%) presented with a rhythm of VF or VT and 34 (61.8%) survived to hospital discharge. Patients presenting with non-shockable rhythms accounted for 10 (18.2%) of the 55 cases who underwent PCI with 4 patients surviving to hospital discharge. In those patients with evidence of STEMI on ED arrival, survival after PCI for those with VF/VT was higher than with asystole/PEA (66% vs 0%).

### eCPR Eligibility

Content analysis of each OHCA case file revealed 69 patients who were potentially eligible for eCPR based on epidemiological criteria alone (Fig. 1). Of these, 25 cases were undergoing CPR on arrival to the emergency department with only 17 of these proceeding to have ≥  20 min of resuscitation efforts and thereby falling into the classification of refractory cardiac arrests and reflecting the target study population. Of the grouped cases (*n*  =  69) matched to eCPR 2-CHEER selection criteria without M-CPR, the mean age was 53.75 years with 14 female and 55 male patients. There was a significant predominance of shockable rhythms in this cohort (*n*  =  58). In the true cases of refractory cardiac arrest (*n *   =  17) ROSC was achieved in only 4 cases with no patients surviving to hospital discharge. The mean times from arrest to either ROSC or death being 66.29 min (SD 25.24). Thirteen of these patients (76.4%) presented between “business hours” of 0800—1800.

The mean time of OHCA to ROSC was 8.73 min (SD 15.67); timing of OHCA to hospital arrival was 47.13 min (SD 21.57) and time from CPR initiation to termination in non-survivors was 79.7 min (SD 27.53).

In cases who did not fulfil eCPR 2-CHEER criteria (*n*  =  179), there was a male gender predominance (74.3%) with a mean age 66.2 years. Of these 49.1% (*n*  =  88) had reported ROSC with 12.2% (*n*  =  22) surviving to hospital discharge. The most common reason for exclusion from the 2-CHEER criteria was age (*n*  =  95) accounting for 53.1% of exclusions. Absence of bystander CPR excluded 36.3% of patients (*n*  =  65) from qualification. A further 49 cases were excluded as ‘unwitnessed’ arrests. Presenting rhythm exclusion criteria was observed in 50 cases. All criteria for exclusion are listed in Table 4.

**Table 4:**
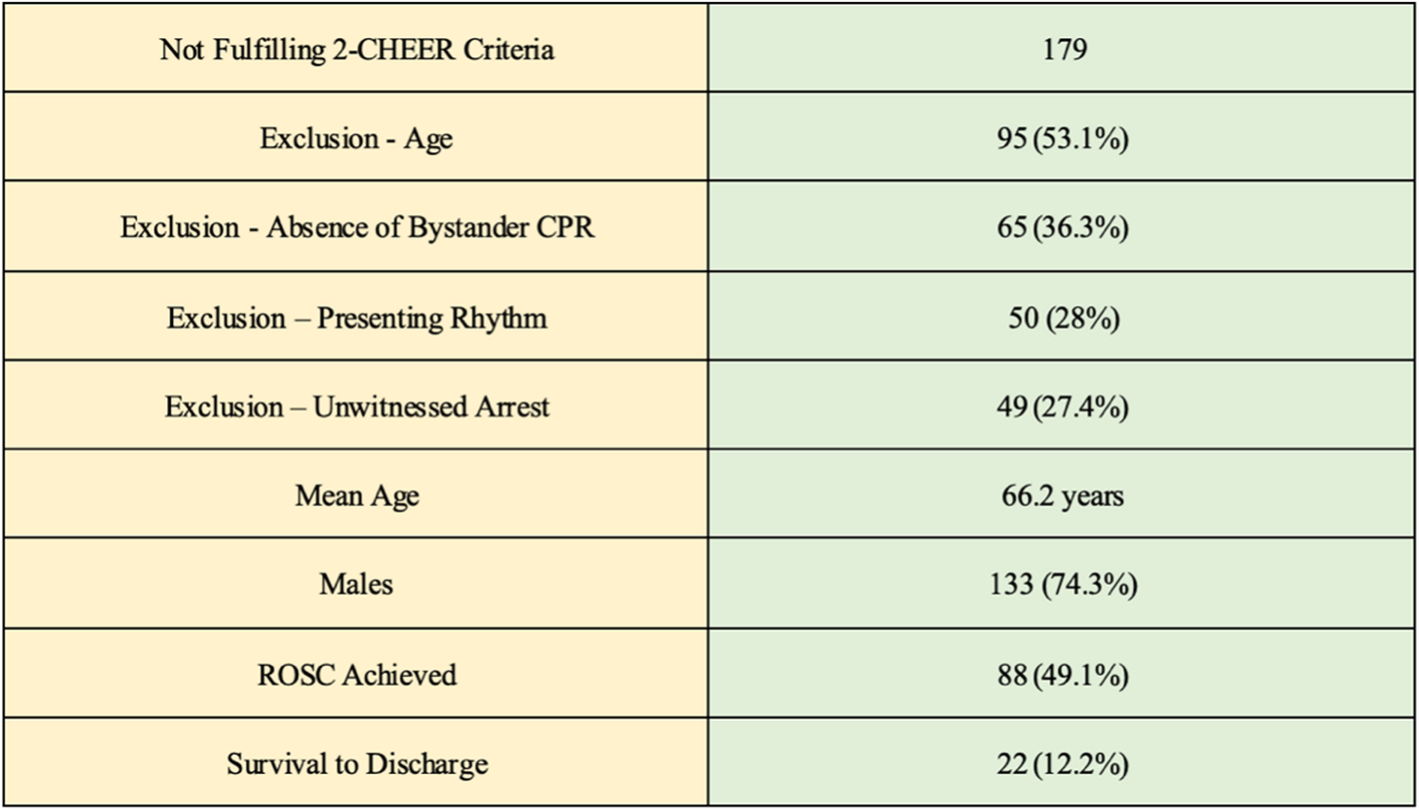
Characteristics of excluded patients (2—CHEER) criteria

## Discussion

Our primary goal in this study was to estimate future caseloads of ECPR in Western Sydney and to facilitate whole of hospital planning in the provision of the service, acknowledging that the involvement of all critical care services and cardiology was essential. Case estimation was performed by matching each case from our OHCA cohort with the contemporary evidence-based eCPR eligibility criteria. Overall, we matched 69 OHCA cases to the 2-CHEER study criteria. 17 of these were true refractory OHCA cases with all but two presenting within regular office hours. These cases represent a significant portion (6.8%) of baseline OHCA presentations in a location without a history of ECPR or prehospital MCPR use. In systems where prehospital MCPR and ECPR are available this may lead to changes in the total number of refractory arrests presenting to the ED [19]. True refractory OHCA cases (defined as failure to achieve ROSC despite 20 min of ALS resuscitation) appear to represent the group most likely to benefit most from eCPR, no patients of this nature in this study survived to hospital discharge [18]. ECPR use in this setting may offer a management option to bridge patients to definitive treatment.

The reported literature reflects a growing use of ECPR in conjunction with an expansion in clinical indications globally but transitioning from standard ALS to ECPR is likely to be a challenge to existing resources [20]. In addition to skilled practitioners and training the use of hospital wide and intensive care unit (ICU) resources will be required, including management of reported complications including cannulation related complications, limb ischemia, acute kidney injury, hemorrhage and stroke [21].

It is conceivable that as awareness of ECPR increases and pre-hospital MCPR is implemented that a larger number of eCPR eligible cases may present to the ED along with a possible increase of eCPR ineligible patients as well [22]. The systems level implications of a structured MCPR to ECPR process is yet to be elucidated. Furthermore, the most appropriate model to maximize “reach” of the service whilst balancing cost effectives and resource capabilities remains unclear. International experience has demonstrated the emergence of a variety of models of care around the provision of a cost effective ECMO services involving multidisciplinary training within appropriate clinical spaces which demonstrates improvements in patient care and fiscal outcomes [23].

Pre-hospital prognostication and management of OHCA is often challenging [24]. Although evidence is available on how to prognosticate OHCA patients, no specific factor universally predicts survival. Therefore, there is controversy on which patients should be eligible for ECPR studies. Regarding the role of prehospital services three specific parameters were assessed in this study including time from arrest to ROSC, time from arrest to time to triage and mean time from arrest to time of the patient being declared deceased. Our collected data for reported mean times of arrest to ROSC and mean transport times is reflective of our excellent baseline prehospital service capability and demonstrates potential to further integrate care with hospital teams. To this end further optimising transportation to hospital with ongoing ALS in refractory cases is required in order to deliver ECPR within current eligibility guidelines. Working with our ambulance colleagues in this area is a cornerstone of delivering a truly interdisciplinary and holistic OHCA response. This interface is particularly pertinent when considering the intervention of ECPR which in current guidelines includes the prehospital initiation of MCPR, an element that was not reviewed in our data. The future availability of this technology and the extent to which it is adopted by ambulance services would significantly impact the viability of an ECPR service.

Additionally, in this study we assessed Utstein-based OHCA outcomes in a contemporary cohort of Australian patients. The key descriptive data analysed was in keeping with the reported literature [3]. We observed that survival rates were higher in patients presenting shockable rhythms, witnessed arrests and with bystander CPR. Further it was observed that survival was higher compared to local and historical reporting of OHCA outcomes. This unexpected finding may be anomalous due to a selection bias in this ED cohort associated with paramedic protocols allowing CPR cessation in the field [25].

Of particular interest is the importance of immediate effective bystander CPR, reflected by the survival outcomes in this study without bystander CPR (16.6%) versus with bystander CPR (30.5%). Future local work must focus on strategies to reduce the apparent high proportion of patients who do not receive bystander rescue (27.8%, *n*  =  65). A recent relevant meta-analyses suggested community based interventions are efficacious in improving rates of bystander CPR [26]. Population surveys indicated only 22% of the Australian public are trained in CPR provision, with these numbers remaining unchanged over the last decade [27], and is low by international standards. Concerted education and training programs have shown significant benefits in improving bystander CPR rates [28]. Local efforts have begun that will hopefully provide improvements [29]. The likelihood of initiating high-quality CPR has been shown to increase even after very brief community-based education programs focused on basic life support [30]. Improving the quality of bystander and prehospital care of OHCA victims holds the key to driving incremental improvement in survival as well as increasing the number of patients eligible for eCPR in the event of a refractory OHCA. Public-health based approaches should be viewed as the cornerstone of an integrated approach to improving resuscitation outcomes [3, 31].

## Limitations

The major limitations of this study relate to the specific single-centre urban location limiting generalisability. Furthermore case load estimation is obtunded by the exclusion of patients in whom resuscitation efforts were ceased in the field due to a lack of ROSC. Local ambulance services employ a termination of resuscitation criteria that is implemented on a case by case basis but involves the cessation of resuscitation efforts in the absence of ROSC or the presence of a non shockable rhythm after 20 min of resuscitation attempts. It is unclear how many of these patients would potentially be included in the context of the prehospital M CPR use. However, given that the case load estimation was performed in a system without the current use of prehospital M-CPR the inclusion of these patients would be as yet untenable within the bounds of the current system. They do however represent a methodological flaw within the derived data set. In addition observational studies are associated with inherent biases and the data extraction in the study was from multiple sources (prehospital, ED and ICU). Therein, it is possible that clerical errors in the original data entry may in turn have led to inaccuracies in our results. Improving OHCA outcomes requires not only a co-ordinated multidisciplinary approach within hospital-based models of care, but also requires liaison with all stakeholders and services.

Two potential limitations pertain to the methodological flaws of this study and possibly effect the estimation of potential caseload numbers. The limitation of excluding non-transported patients and the single centre nature of the study potentially missing patients who were transported to other facilities within the area health service presents a selection bias that likely would cause an underestimation of true case numbers. Within those non transported patients it should also be noted that pre hospital services use termination of resuscitation criteria that might further effect the integrity of the larger data set. The implementation of M-CPR into the prehospital service provision may cause an increase in the number of presentations for ECMO applicable patients and may in fact cause an increase in the presentation of patients in general to centres that offer ECMO services. Appropriate inclusion criteria, the provision of training and patient support resources (social workers etc.) and the use of diagnostic investigations in patients on ECMO all require resource allocation and focus as part of the the planning from a systems perspective [27].

## Conclusion

The results reflect a higher than traditionally expected OHCA survival rate in a contemporary cohort of ED patients. At baseline more than 5% of OHCA patients may be eligible for eCPR based on the 2-CHEER criteria. The demonstrated low survival in those patients deemed to have “refractory” cardiac arrests further attests to the potential benefits that may be gained from the implementation and use of eCPR in a coordinated and well-resourced system and in conjunction with public health education to improve rates and quality of bystander CPR in the community. Further studies and cost benefit analysis are needed to identify where future local quality improvement strategies should be focused.

## Data Availability

The datasets generated during and analysed during the current study are not publicly available due to containing sensitive demographic data but are available from the corresponding author on reasonable request.

## Abbreviations

OHCA: Out of hospital cardiac arrest
eCPR: Extracorporeal cardiopulmonary resuscitation
ECMO: Extracorporeal membrane oxygenation
ROSC: Return of spontaneous circulation
ALS: Advanced life support
m-CPR: Mechanical CPR
LHD: Local health district
VF: Ventricular fibrillation
VT: Ventricular tachycardia
PCI: Percutaneous coronary intervention
